# Evaluation of Microleakage of Orthograde Root-Filling Materials in Immature Permanent Teeth: An In Vitro Study

**DOI:** 10.1155/2024/8867854

**Published:** 2024-10-29

**Authors:** Upma Das, Vanita Gautam, Snigdha Shubham, Shristi Raut

**Affiliations:** ^1^Department of Conservative Dentistry and Endodontics, Universal College of Medical Sciences, Bhairahawa, Nepal; ^2^Department of Microbiology, Universal College of Medical Sciences, Bhairahawa, Nepal

**Keywords:** adseal, apical microleakage, biodentine, dye penetration, fluorescence, MTA, MTA plus, open apex, root-end filling

## Abstract

**Introduction:** The absence of a barrier in an open root apex makes endodontic treatment challenging as root-filling material can easily reach the surrounding tissue. The aim of the study was to compare the apical microleakage associated with mineral trioxide aggregate (MTA), biodentine, custom-made gutta-percha with MTA plus and custom-made gutta-percha with Adseal in immature permanent teeth by dye penetration method.

**Methods:** Apical 2 mm of 60 single-rooted mandibular premolar teeth was resected to create divergent open apices and 10 teeth each were filled with Biodentine plug, MTA plug, custom-made gutta-percha with MTA plus sealer and custom-made gutta-percha with Adseal sealer. Ten teeth each acted as positive and negative controls. All the samples were stored at 37°C at 100% humidity for 5 weeks and then immersed in 2% Rhodamine B dye for 24 h. Transverse sectioning was done apically at 1 mm and 3 mm to evaluate dye penetration under a fluorescence microscope using ImageJ software.

**Results:** There was a significantly greater microleakage at 1 mm cross section compared to 3 mm (*p* < 0.0001). At 1 mm cross section, the apical microleakage was the highest for the MTA plug with a mean leakage percentage of 48.08 ± 16.38, a mean depth of leakage of 0.46 ± 0.10 mm and a mean area of leakage of 1.35 ± 0.74 mm^2^, compared to other groups, and the difference was statistically significant. However, at a 3 mm cross section, MTA plus sealer with gutta-percha demonstrated the highest mean leakage percentage (25.01 ± 7.77) compared to other groups and the difference was statistically significant (*p* = 0.03).

**Conclusion:** It can be concluded that the 3-mm-thick apical plug provided better sealing of the open apex compared to the 1 mm apical plug and there was no significant difference in microleakage among the Biodentine plug, MTA plug and Adseal sealer with gutta-percha plug at 3 mm cross section.

## 1. Introduction

The most crucial aspect of successful endodontic treatment is the complete sealing of the root canal system with a biologically inert material [1]. This involves removing the infected content of the canal and filling the root canal system three-dimensionally within the root's confines. Typically, the root canal of a tooth extends from the canal's orifice to the apical foramen. The apical region of the root canal consists of three parts: the apical constriction (the narrowest part of the canal before the foramen), the major diameter (which is almost twice the diameter of the apical constriction, giving it a funnel shape) and the cemento-dentinal junction (where cementum meets dentin). The apical constriction is typically recommended as the ideal place to stop the root canal filling [2]. However, some teeth may not have an apical constriction due to various reasons, such as pulp necrosis during root development or incomplete root formation caused by trauma, deep caries or other pulpal conditions. Open apices can also occur due to apical resorption or iatrogenic events [3, 4].

Obtaining a complete seal in a tooth with an open apex is a challenging task. The aim of cleaning and shaping the root canal is to create enough space to hold the filling material, which can resist the internal compressive forces of obturation and prevent the filling material from extruding out of the canal. Without proper resistance, it is difficult to achieve a satisfactory seal at the apex and prevent the filling material from extending beyond the canal [5]. The presence of moisture in an open apex further adds to the difficulty in achieving a good seal. Inadequate sealing can result in harmful tissue irritants and bacteria entering or exiting the canal, ultimately affecting the success of the treatment in the long term [6].

Different endodontic approaches have been developed to achieve a complete seal in immature apices with open apex, such as using custom-made gutta-percha filled with sealers like zinc oxide or bioceramic-based materials that adapt closely to the root canal wall [7]. While calcium hydroxide (CH) has traditionally been preferred for its favourable biological performance, it requires multiple treatment sessions and temporary coronal restorations that can be vulnerable to reinfection. Additionally, prolonged exposure to CH can lead to thin roots and increase the likelihood of tooth fracture [8]. Mineral trioxide aggregate (MTA) has been found to be an effective alternative to CH for apexification, with success rates ranging from 77% to 100% [9–12]. Endodontic treatment for immature teeth with open apex varies depending on the case selection criteria, dental pulp status as vital or nonvital or irreversible pulpitis with a bad prognosis for continued vitality and the development stage of maturity of the root canal. Apexification is the most desirable endodontic treatment for immature teeth with open apex and necrotic pulps where root dentinal walls are thick and can resist fracture. However, procedures such as revascularization are considered for nonvital immature permanent teeth with open apex and thin dentinal walls, especially in patients aged 6–9 years, as it helps in dentin formation and strengthens the weak root structure [13]. Recently, new calcium silicate–based root-end filling materials have been introduced, showing good sealing properties, biocompatibility and increased resistance to fracture [14].

Although newer techniques for root-end filling in endodontics have been developed, achieving a complete seal of an immature tooth with an open apex using orthograde or retrograde techniques is still a challenge. Apical leakage is the primary reason for endodontic failure, and it is affected by various factors such as root canal filling techniques, physical and chemical properties of root-end filling materials and the presence or absence of a smear layer [15].

Several studies have assessed the sealing ability of different materials to manage open apices in immature permanent teeth using various methods such as bacterial leakage [16], fluid filtration [17], radioisotope penetration [18], dye extraction [19] and micro-CT imaging [20]. Most of these studies were performed on simulated immature apices where divergent open apices were created by Gates Glidden drill or Ni-Ti files using various calcium silicate base cement [21–23]. However, there is a lack of sufficient research regarding the treatment outcome of orthograde treatment with the most effective root-end filling material [24]. Therefore, the objective of this study was to identify the ideal orthograde root-end filling material in the apical third of the immature permanent teeth by assessing the microleakage of four different materials, namely MTA plug, Biodentine plug, custom-made gutta-percha with MTA plus sealer and custom-made gutta-percha with Adseal sealer. The alternate hypothesis was, there is significant difference in apical microleakage between MTA plug, Biodentine plug, custom-made gutta-percha with MTA plus sealer and custom-made gutta-percha with Adseal sealer at 1 mm and 3 mm cross section.

## 2. Materials and Methods

This in vitro study was conducted in the Department of Conservative and Endodontics after obtaining ethical approval (Reference number: UCMS/IRC/219/19) from the Institutional Review Committee of Universal College of Medical Sciences, Bhairahawa, Nepal. Sixty intact single-rooted premolars of approximately 22.5 ± 2 mm in length, which were extracted for orthodontic treatment, were selected for this study. The required sample size was determined using G^∗^Power 3.1.9 (Universität Kiel, Kiel, Germany) to identify significant differences (*α* = 0.05, 80% power), resulting in an anticipated sample size of 10 teeth within each group. Caries-free teeth without signs of fractures or cracks and with similar lengths were included in the study. Teeth with immature root apices, thin curved roots, restoration, hypocalcification or hypoplasia, the presence of multiple or lateral canals, calcified root canal, and severe apical curvature and with more than one root or canal were excluded. Immediately after extraction, the teeth were immersed in 5.25% of sodium hypochlorite (Prime Dental, SM Dental Care, New Delhi, India) for 5 min and an ultrasonic scaler (Woodpecker, SM Dental Care, New Delhi, India) was used to remove soft tissue debris, calculus and external stain from the teeth before the examination of crack, caries and fracture under illumination. According to the guidelines of CDC, the extracted teeth should be sterilized by autoclaving or storage in 10% formalin before using for educational or research purposes [25]. Samples were then stored in a 10% buffered solution of Formalin until further use (Supporting document 1).

### 2.1. Preparation of Samples

Each tooth was resected 2 mm at the root apex (Supporting document 1), and a divergent open apex was created by retrograde use of orifice enlarger #25 0.08% taper rotary file of Hyflex CM (Coltene/Whaledent Inc., USA) inserted to the length of cutting blade *D* = 16 by a single operator as per the method described by Hachmeister et al. [26]. The final apical diameter was 0.8 mm. An access cavity preparation was done with Endoaccess Bur (Dentsply, India), and the canal orifice was located with DG16 (GDC, India) explorer. The patency of the canal was checked with the help of a 15 k file, and the working length was taken 0.5 mm shorter than the divergent open apex as confirmed with Radiovisiography (RVG) (Supporting document 1).

With the estimated working length and in the presence of Glyde (Dentsply, India), a glide path was established. Initially, cleaning and shaping of the canal was done up to 20 k file, and then, 0.04% taper #40 Hyflex CM (Coltene/Whaledent, Inc. USA) file was used to prepare the canal. Irrigation of the canal was done with 5 mL of 5.25% of sodium hypochlorite, 3 mL of 17% of EDTA and 5 mL of saline. After the preparation of the canal, the samples were embedded in a wet sponge to simulate the periapex and to prevent the extrusion of filling material out of the apex.

The samples were randomly divided into six groups with 10 specimens in each group (Group I—Biodentine plug, Group II—MTA plug, Group III—MTA plus sealer with custom-made gutta-percha, Group IV—Adseal sealer with custom-made gutta-percha, Group V—Positive control and Group VI—Negative control). The root canals were irrigated with normal saline and dried with paper points. All teeth were coated with two coats of nail varnish leaving 1 mm at the apex except the negative control group where all surfaces were coated with two coats of nail varnish. In addition, root canals were not filled in both positive and negative control groups (Supporting document 2). Out of four experimental groups, the first group was filled with 5 mm of Biodentine plug (Septodont Inc., France) and the remaining part of the canal was filled with gutta-percha (Diadent, India) and zinc oxide eugenol sealer using lateral condensation technique and sealed with glass ionomer cement (Shofu, China). The second group was filled with 5 mm of ProRoot MTA plug (Dentsply, India), and the remaining part of the canal was filled with paper point and sealed with Cavit (3M ESPE). After 2 days, the paper point was removed and setting of the material was tested with paper point pressure. The final obturation was done with gutta-percha and zinc oxide eugenol sealer using lateral condensation technique and restored with glass ionomer cement (Shofu, China). The third group was obturated with custom-made gutta-percha with MTA plus (Prevest, India) sealer, and the fourth group was obturated with custom-made gutta-percha and Adseal (Meta, Republic of Korea) sealer. The custom-made gutta-percha was made by heating two or more, large gutta-percha cones and rolling them together between two glass slabs to obtain an appropriate size [2]. Radiographs were taken to verify proper fit and position of gutta-percha. All materials were mixed according to the manufacturer's instructions. Finger pluggers (Dentsply, India) were used for the vertical condensation of root-filling materials in the canal (Supporting document 2). The adequacy of root fillings was confirmed and evaluated under RVG.

### 2.2. Dye Penetration

The apex of each specimen was immersed in 2% of Rhodamine B dye (Sisco Research Laboratories, India) at room temperature in Eppendorf tubes for 24 h (Supporting document 3). After 24 h, the samples were washed under running tap water to remove the traces of dye. The teeth were dried and the nail varnish was removed with the help of a Lecron carver, and the specimens were sectioned at 1 mm and 3 mm from the apex with the diamond disc bur and viewed at 100x magnification under a fluorescence microscope (Zeiss, Germany) (Supporting document 3).

### 2.3. Microscopic Evaluation

**Figure 1:**
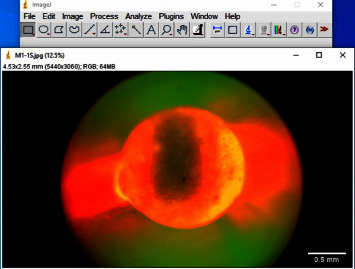
Sample image imported to ImageJ software.

**Figure 2:**
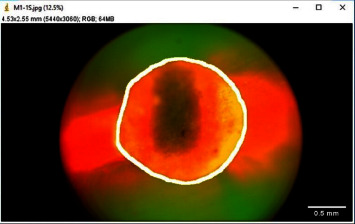
Measurement of canal circumference.

**Figure 3:**
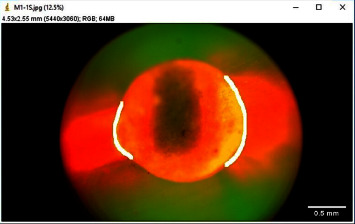
Measurement of leakage circumference.

The images were imported to ImageJ software (Wayne Rasband and contributors, National Institute of Health, USA) (with Java 1.8.0_172) for analysis (Figure 1). For all images, first of all, the canal circumference was measured (Figure 2). Then, the circumference of the canal displaying leakage was measured (leakage circumference—Figure 3). After that, the leakage percentage was calculated using the following formula:

(1) $$\begin{array}{c} \text{Leakage percentage}=\frac{\text{Leakage circumference}}{\text{Canal circumference}}\times 100\%. \end{array}$$

**Figure 4:**
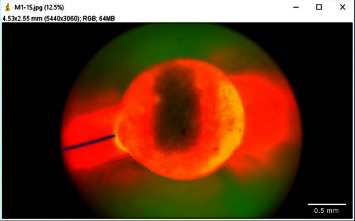
Measurement of depth of leakage.

**Figure 5:**
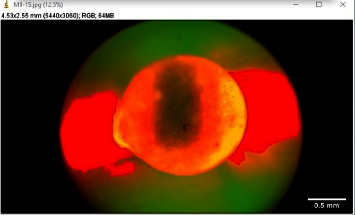
Measurement of area of leakage.

The leakage percentage thus calculated was graded according to the degree of leakage as follows: Grade 0: 0% leakage, Grade 1: < 25% leakage, Grade 2: 25%–50% leakage, Grade 3: 50%–75% leakage and Grade 4: > 75% leakage [14]. Subsequently, the depth of leakage (Figure 4) and the area of leakage (Figure 5) present in the image at 100x magnification were measured [27, 28].

### 2.4. Statistical Analysis

The data were entered in a Microsoft Excel sheet and transferred to Statistical Package for Social Sciences software (IBM SPSS Statistics for Windows, Version 20.0. Armonk, NY: IBM Corp) for analysis. Descriptive statistics such as frequency distribution, mean and standard deviations were calculated for the samples. In the inferential statistics, an independent sample *t*-test was used to compare the means between the samples at 1 mm and 3 mm cross sections. Likewise, one-way ANOVA and post hoc Tukey test were used to compare the means of four groups, i.e., Group I (Biodentine plug), Group II (MTA plug), Group III (MTA plus sealer with custom-made gutta-percha) and Group IV (Adseal sealer with custom-made gutta-percha) at both 1 mm and 3 mm cross sections. The *p* value for the level of significance was set at 0.05 with a 95% confidence interval.

## 3. Results

**Figure 6:**
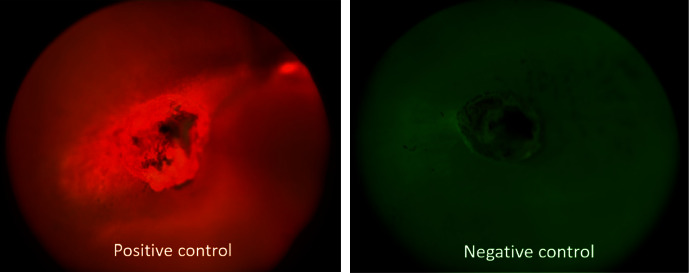
Images showing microleakage in positive and negative controls.

The degree of microleakage for all six groups, namely Group I (Biodentine plug), Group II (MTA plug), Group III (MTA plus sealer with custom-made gutta-percha), Group IV (Adseal sealer with custom-made gutta-percha), Group V (Positive control) and Group VI (Negative control), was assessed using dye penetration technique at 1 mm and 3 mm cross sections. All the positive controls demonstrated 100% microleakage, and all the negative controls demonstrated 0% microleakage in both 1 mm and 3 mm cross sections. Hence, it was not practical to compare them with the remaining four groups; therefore, positive and negative controls were not subjected to further statistical analysis (Figure 6).

All the samples of the remaining four groups (Biodentine plug, MTA plug and MTA plus sealer with custom-made gutta-percha and Adseal sealer with custom-made gutta-percha) displayed some degree of microleakage at 1 mm cross section, and none of the samples had 0% microleakage (Table 1); however, 13 samples at 3 mm cross section had 0% microleakage, out of which seven belonged to Biodentine apical plug group and three each belonged to MTA apical plug group and Adseal sealer with custom-made gutta-percha group, whereas MTA plus sealer with custom-made gutta-percha had zero samples without leakage (Table 2).

The mean leakage percentage, depth of leakage and area of leakage were higher at 1 mm cross section compared to 3 mm cross section, and the difference was found to be statistically significant (Table 3).

For the cross section at 1 mm, the mean leakage percentage, depth of leakage and area of leakage were highest for the MTA plug group and the lowest for the Biodentine plug group. Furthermore, one-way ANOVA revealed a statistically significant difference in all three parameters among the groups (Table 4).

Pairwise comparison using the post hoc Tukey test revealed that the mean leakage percentage and area of leakage for the MTA plug group were significantly higher than those of the Biodentine plug, MTA plus sealer with custom-made gutta-percha and Adseal sealer with custom-made gutta-percha. However, there was no statistically significant difference between the mean leakage percentage and area of leakage of the Biodentine plug, MTA plus sealer with custom-made gutta-percha and Adseal sealer with custom-made gutta-percha groups. Furthermore, the Tukey test revealed that the mean depth of leakage for the MTA plug group was significantly higher than that of the Biodentine plug group. However, there was no statistically significant difference among the Biodentine plug, MTA plus sealer with custom-made gutta-percha and Adseal sealer with custom-made gutta-percha groups in terms of depth of leakage.

For the cross section at 3 mm, the mean leakage percentage was the highest for MTA plus sealer with custom-made gutta-percha group and the lowest for the Biodentine plug group. One-way ANOVA revealed a statistically significant difference in mean leakage percentage among the groups. Therefore, a pairwise comparison between the groups was done by the post hoc Tukey test. The Tukey test revealed that the mean leakage percentage for MTA plus sealer with custom-made gutta-percha group was significantly higher than those of the Biodentine plug, MTA plug and Adseal sealer with custom-made gutta-percha groups. However, there was no statistically significant difference among the Biodentine plug, MTA plug and Adseal sealer with custom-made gutta-percha groups. Interestingly, there was no statistically significant difference between the mean depth of leakage and mean area of leakage among the groups at a 3 mm cross section (Table 5).

## 4. Discussion

This in vitro study was conducted to evaluate the apical microleakage of orthograde MTA plug, Biodentine plug, MTA plus sealer with custom-made gutta-percha and Adseal sealer with custom-made gutta-percha at 1 mm and 3 mm cross sections from the root apex in simulated immature apex by Rhodamine B dye penetration under a fluorescent microscope. Under the experimental condition, the results showed that all the samples of the four groups displayed some degree of microleakage at 1 mm cross section; however, 13 samples at 3 mm cross section had 0% microleakage.

While comparing the leakage in terms of leakage percentage, area of leakage and depth of the leakage in cross sections at 1 mm and 3 mm, it was found that the value of leakage was higher at 1 mm compared to 3 mm for all four groups. This finding is similar to that of Bani, Sungurtekin-Ekçi and Odabaş where the amount of microleakage was significantly lower for 3 mm and 4 mm apical plugs compared to 1 mm and 2 mm subgroups of Biodentine and MTA [29]. Similarly, it was also reported that a 3-mm- to 5-mm-thick MTA plug produces a reasonable seal that improves with time in the presence of phosphate-containing solution [30]. Another study found that 1 mm thickness was the least effective in preventing apical leakage while 4 mm thickness of MTA was better in preventing leakage [31]. Furthermore, Khullar et al. observed the deepest tubular penetration at 5 mm from the apex than at a 3 mm level of the root canal for Adseal and BioRoot RCS sealers [32].

The rationale for the greater leakage at 1 mm compared to 3 mm is due to the thickness of the apical plug, i.e., the apical plug of a greater thickness of up to 5 mm provides a better seal than that of 1 mm. Secondly, in the case of customized gutta-percha with sealer, the more the penetration of the sealer into the dentinal tubules, the greater will be the sealing ability. As the number and diameter of dentinal tubules decrease apically in the root canal, it leads to lesser penetration of sealer into dentinal tubules in the apical region, so at 1 mm of the apex where there was a lack of sealer penetration, dye has easily penetrated into that area and resulted in more leakage [33]. Furthermore, inherent irregularities, the divergent nature of the apical anatomy and limited condensation due to minimal resistance of the open apex affect the adaptation of root-end filling materials to dentin walls [26].

In this study, at 1 mm cross section, the mean leakage percentage, depth of leakage and area of leakage were the highest for the MTA plug group and the lowest for the Biodentine plug group. The result of this study was similar to the result of Nanjappa et al. who found that Biodentine had a better sealing ability than MTA [34]. It was also found that Biodentine showed the least amount of microleakage as compared with MTA and glass ionomer cement when a dye leakage study was performed [35]. Biodentine also exhibited less microleakage than ProRoot MTA, when compared using the dye penetration technique [36]. When root-end filling materials for margin adaptation were compared using a confocal laser scanning microscope, it was found that Biodentine had better marginal adaptation than MTA and glass ionomer cement [37]. Studies have found that Biodentine exhibits superior sealing properties than MTA [38, 39]. When the marginal adaptation of MTA, Biodentine and MTA plus was evaluated by scanning electron microscope, Biodentine showed the least gap at the interface, whereas MTA plus and ProRoot MTA had similar interfacial gaps [40]. However, recent clinical studies have shown similar sealing ability between MTA and Biodentine, although the treatment time was shorter with Biodentine [41, 42]. Similarly, a study found that the use of collagen as an apical barrier prior to the MTA plug was associated with favourable clinical outcomes [43].

A study reported that the use of MTA as an orthograde filling material produced a significantly higher percentage of voids than did gutta-percha [44]. Moreover, with MTA, several voids were observed in the apical region. MTA exhibited significantly poorer sealing quality in root canals with complex canal types; however, this difference was not observed in the root canals selected for this study [44].

However, unlike our study, in various studies, MTA has demonstrated less microleakage compared to other materials. Bani, Sungurtekin-Ekçi, and Odabaş and Butt et al. reported contradictory results to those in our study, and they observed no significant difference between the microleakage value of MTA and Biodentine with Biodentine exhibiting similar properties to MTA [29, 45]. Comparative studies between MTA and Biodentine revealed that both materials offer excellent sealing performance which prevents the risk of subsequent microbial contamination [46].

In the present study, there was a significant difference in the leakage value of the MTA plug and Biodentine plug, showing that MTA has more leakage than Biodentine. This could be due to the faster setting time of Biodentine which prevents bacterial infiltration and microleakage. The smaller particle size and wider calcium and silicate-rich dentine area and longer incorporation depths of Biodentine into dentine also allow better marginal adaptation and sealing ability as compared to MTA [47].

Our finding that the MTA plug group had a greater degree of microleakage as compared with the Adseal sealer with customized gutta-percha group is in agreement with Sreedev [48]. The reason behind less microleakage in Adseal is due to the property of epoxy resin base sealer to react with any exposed amino group in collagen when the epoxy ring is open. This bond is maintained by low polymerization stress, long-term dimensional stability and efficient cohesion between molecules [48]. Similarly, Singh et al. demonstrated the better sealing of Adseal by explaining the fact that epoxy resin-based sealer is considered contraction-free during setting reaction which is responsible for its interfacial adaptation [49].

In this study, at a 3 mm cross section, MTA plus revealed the highest leakage percentage in comparison with other groups. The result of the present study was in accordance with Ballullaya et al. that MTA-based sealers have excellent biological properties, but the MTA plus sealer does not bond either with dentin or gutta-percha, which may be the reason for increased microleakage. Because of the short working time, MTA plus sealer results in an improper coating of the canal during subsequent lateral compaction. Also, the poor sealing ability of MTA plus might be due to the presence of voids in two interfaces of the sealer [50]. Unlike our result, Saraswathi et al. and Bansal et al. demonstrated a better sealing ability and lesser microleakage with MTA plus [40, 51]. However, it is worth pointing out that MTA plus was used as a plug, not as a sealer in those studies.

Apical microleakage is the primary reason for endodontic treatment failure in nonvital immature apices. Various techniques, such as dye penetration and fluid filtration, are used to assess root-end filling microleakage [52]. In this study, microleakage was evaluated by dye penetration technique using Rhodamine B dye under a fluorescent microscope. This method is considered easy and useful since it shows the extent of the gap between the root canal wall and the root-filling material [53]. Additionally, various tracer solutions can be used in this technique, such as methylene blue, silver nitrate dye, Rhodamine B dye, India ink, Basic fuchsin and others. Rhodamine B dye was used in this study to evaluate microleakage due to its high fluorescence and water solubility [54].

There are a few limitations to this study. While this study aimed to identify the most effective root-end filling material by comparing apical microleakage among various materials, the ultimate choice also considers factors like biocompatibility, osteoinduction and osteoconduction. It is worth noting that MTA and Biodentine have demonstrated superior biocompatibility, osteoinduction and osteoconduction [55–57]. This was an in vitro study, so the results could differ in the oral environment—an in vivo study is therefore required. Dye penetration extent could be affected by negative or positive pressure, and this might have affected our results. Sectioning of teeth is a technique-sensitive procedure and may result in the loss of part of the teeth while sectioning. Though the sectioning was done diligently, the loss of part of teeth could not be avoided in all cases.

## 5. Conclusions

This study found that the apical microleakage was significantly higher at the 1 mm cross section compared to the 3 mm cross section. Overall, a 3-mm apical plug offered better sealing, and there was no significant difference among Biodentine, MTA and Adseal at 3 mm, suggesting they are all suitable choices for clinical use.

## Figures and Tables

**Table 1:** Distribution of samples according to leakage grading among the groups at 1 mm.

Grading of leakage	Biodentine plug	MTA plug	MTA plus sealer with GP	Adseal sealer with GP	Total	Percent
0	0	0	0	0	0	0
1	6	0	1	7	14	35
2	4	6	9	3	22	55
3	0	2	0	0	2	5
4	0	2	0	0	2	5
Total	10	10	10	10	40	100

Abbreviations: GP = custom-made gutta-percha, MTA = mineral trioxide aggregate.

**Table 2:** Distribution of samples according to leakage grading among the groups at 3 mm.

Grading of leakage	Biodentine plug	MTA plug	MTA plus sealer with GP	Adseal sealer with GP	Total	Percent
0	7	3	0	3	13	32.5
1	1	4	5	5	15	37.5
2	2	2	5	2	11	27.5
3	0	1	0	0	1	2.5
4	0	0	0	0	0	0
Total	10	10	10	10	40	100

Abbreviations: GP = custom-made gutta-percha, MTA = mineral trioxide aggregate.

**Table 3:** Comparison of microleakage at 1 mm and 3 mm.

Parameters	Cross section (mm)	Number of samples	Mean ± SD	*t*	*p* value
Leakage percent (%)	1	40	30.91 ± 8.35	4.43	<0.0001
	3	40	16.14 ± 4.42		

Depth of leakage (mm)	1	40	0.35 ± 0.14	4.68	<0.0001
	3	40	0.19 ± 0.06		

Area of leakage (mm^2^)	1	40	0.63 ± 0.06	3.74	<0.0001
	3	40	0.15 ± 0.02		

Abbreviations: % = percentage, mm = millimetres, mm^2^ = millimetre square, MTA = mineral trioxide aggregate, SD = standard deviation.

**Table 4:** Comparison of apical microleakage among the groups at 1 mm.

Parameters	Groups	*N*	Mean values	*F*	*p* value
Leakage percentage (%)	Biodentine plug	10	21.42 ± 8.96	13.01	<0.001
	MTA plug	10	48.08 ± 16.38		
	MTA plus sealer with custom-made gutta-percha	10	32.73 ± 8.38		
	Adseal sealer with custom-made gutta-percha	10	21.54 ± 8.44		

Depth of leakage (mm)	Biodentine plug	10	0.29 ± 0.09	3.09	0.039
	MTA plug	10	0.46 ± 0.10		
	MTA plus sealer with custom-made gutta-percha	10	0.35 ± 0.13		
	Adseal sealer with custom-made gutta-percha	10	0.31 ± 0.18		

Area of leakage (mm^2^)	Biodentine plug	10	0.12 ± 0.09	20.04	<0.001
	MTA plug	10	1.35 ± 0.74		
	MTA plus sealer with custom-made gutta-percha	10	0.36 ± 0.12		
	Adseal sealer with custom-made gutta-percha	10	0.21 ± 0.11		

Abbreviations: % = percentage, mm = millimetres, mm^2^ = millimetre square, MTA = mineral trioxide aggregate, *N* = sample size.

**Table 5:** Comparison of apical microleakage among the groups at 3 mm.

Parameters	Group	*N*	Mean values	*F*	*p* value
Leakage percentage (%)	Biodentine plug	10	7.16 ± 1.22	3.33	0.030
	MTA plug	10	19.14 ± 4.96		
	MTA plus sealer with custom-made gutta-percha	10	25.01 ± 7.77		
	Adseal sealer with custom-made gutta-percha	10	13.25 ± 1.16		

Depth of leakage (mm)	Biodentine plug	10	0.21 ± 0.12	2.97	0.054
	MTA plug	10	0.22 ± 0.17		
	MTA plus sealer with custom-made gutta-percha	10	0.24 ± 0.08		
	Adseal sealer with custom-made gutta-percha	10	0.25 ± 0.10		

Area of leakage (mm^2^)	Biodentine plug	10	0.15 ± 0.01	1.61	0.204
	MTA plug	10	0.2 ± 0.02		
	MTA plus sealer with custom-made gutta-percha	10	0.13 ± 0.1		
	Adseal sealer with custom-made gutta-percha	10	0.11 ± 0.09		

Abbreviations: % = percentage, mm = millimetres, mm^2^ = millimetre square, MTA = mineral trioxide aggregate, *N* = sample size.

## Data Availability

The data of this study will be available from the corresponding author at a reasonable request.

## Supporting Information

Supporting Information 1 Supporting document 1: Figure 1: Sample collection and group division, Figure 2: Creation of divergent apex and root canal preparation, and Figure 3: Radiographic image of sample showing divergent apex.

Supporting Information 2 Supporting document 2: Figure 1: Samples with MTA plug, Figure 2: Samples with Biodentine plug, Figure 3: Samples with MTA plus sealer with GP, Figure 4: Samples with Adseal sealer with GP (Group IV), Figure 5: Negative control group, and Figure 6: Positive control group.

Supporting Information 3 Supporting document 3: Figure 1: Immersion of samples in 2% Rhodamine dye and Figure 2: Fluorescence microscope used for examination of sample cross sections.

## References

[1] Schlider H. (1967). Filling Root Canal in Three Dimensions. *Dental Clinics of North America*.

[2] Cohen S., Hargreaves K. M., Keiser K. (2006). *Pathways of the Pulp*.

[3] Flanagan T. A. (2014). What Can Cause the Pulps of Immature, Permanent Teeth With Open Apices to Become Necrotic and What Treatment Options Are Available for These Teeth. *Australian Endodontic Journal*.

[4] Plascencia H., Díaz M., Gascón G. (2017). Management of Permanent Teeth With Necrotic Pulps and Open Apices According to the Stage of Root Development. *Journal of clinical and experimental dentistry.*.

[5] Alani A. H., Toh C. G. (1997). Detection of Microleakage Around Dental Restorations: A Review. *Operative Dentistry*.

[6] Sarnowski A. (2019). *Management of the Open Apex Using a Bioceramic Apical Barrier: Success and Survival Rates at Virginia Commonwealth University*.

[7] Morse D. R., O’Larnic J., Yesilsoy C. (1990). Apexification: Review of the Literature. *Quintessence International*.

[8] Rafter M. (2005). Apexification: A Review. *Dental Traumatology*.

[9] Pace R., Giuliani V., Nieri M., Di Nasso L., Pagavino G. (2014). Mineral Trioxide Aggregate as Apical Plug in Teeth With Necrotic Pulp and Immature Apices: A 10-Year Case Series. *Journal of Endodontics*.

[10] Simon S., Rilliard F., Berdal A., Machtou P. (2007). The Use of Mineral Trioxide Aggregate in One‐Visit Apexification Treatment: A Prospective Study. *International Endodontic Journal*.

[11] Witherspoon D. E., Small J. C., Regan J. D., Nunn M. (2008). Retrospective Analysis of Open Apex Teeth Obturated With Mineral Trioxide Aggregate. *Journal of Endodontics*.

[12] Sarris S., Tahmassebi J. F., Duggal M. S., Cross I. A. (2008). A Clinical Evaluation of Mineral Trioxide Aggregate for Root‐End Closure of Non‐Vital Immature Permanent Incisors in Children‐A Pilot Study. *Dental Traumatology*.

[13] Murray P. E. (2023). Review of Guidance for the Selection of Regenerative Endodontics, Apexogenesis, Apexification, Pulpotomy, and Other Endodontic Treatments for Immature Permanent Teeth. *International Endodontic Journal*.

[14] Hamdan R., Michetti J., Dionnet C., Diemer F., Georgelin-Gurgel M. (2017). In-Vitro Evaluation of Apical Microleakage of Two Obturation Methods of Immature Permanent Teeth: Orthograde Apical Plug of Mineral Trioxide Aggregate and Root Canal Filling Combining Custom Gutta-Percha Cone With Calcium Silicate-Based Sealer. *Giornale Italiano di Endodonzia*.

[15] Muliyar S., Shameem K. A., Thankachan R. P., Francis P. G., Jayapalan C. S., Hafiz K. A. (2014). Microleakage in Endodontics. *Journal of International Oral Health: JIOH*.

[16] Jahromi M., Refaei P., Moughari A. K. (2020). Comparison of the Microleakage of Mineral Trioxide Aggregate, Calcium-Enriched Mixture Cement, and Biodentine Orthograde Apical Plug. *Dental Research Journal*.

[17] Adel M., Salmani Z., Youssefi N., Heidari B. (2021). Comparison of Microleakage of Mineral Trioxide Aggregate Apical Plug Applied by the Manual Technique and Indirect Use of Ultrasonic With Different Powers. *Journal of Dentistry*.

[18] Pereira I. R., Carvalho C., Paulo S. (2021). Apical Sealing Ability of Two Calcium Silicate-Based Sealers Using a Radioactive Isotope Method: An In Vitro Apexification Model. *Materials*.

[19] Tolibah Y. A., Droubi L., Alkurdi S. (2022). Evaluation of a Novel Tool for Apical Plug Formation During Apexification of Immature Teeth. *International Journal of Environmental Research and Public Health*.

[20] Kalaoglu E. E., Duman C., Capan B. S., Ocak M., Bilecenoglu B. (2023). Comparison of Three Different Biomaterials Used in In Vitro Molar Apexification Models. *BMC Oral Health*.

[21] Ghasemi N., Janani M., Razi T., Atharmoghaddam F. (2017). Effect of Different Mixing and Placement Methods on the Quality of MTA Apical Plug in Simulated Apexification Model. *Journal of clinical and experimental dentistry*.

[22] Al-Kahtani A., Shostad S., Schifferle R., Bhambhani S. (2005). In-Vitro Evaluation of Microleakage of an Orthograde Apical Plug of Mineral Trioxide Aggregate in Permanent Teeth With Simulated Immature Apices. *Journal of Endodontics*.

[23] Tran D., He J., Glickman G. N., Woodmansey K. F. (2016). Comparative Analysis of Calcium Silicate–Based Root Filling Materials Using an Open Apex Model. *Journal of Endodontics*.

[24] Torabinejad M., Corr R., Handysides R., Shabahang S. (2009). Outcomes of Nonsurgical Retreatment and Endodontic Surgery: A Systematic Review. *Journal of Endodontics*.

[25] Western J. S., Dicksit D. D. (2016). A Systematic Review of Randomized Controlled Trials on Sterilization Methods of Extracted Human Teeth. *Journal of Conservative Dentistry*.

[26] Hachmeister D. R., Schindler W. G., Walkeriii W., Deneethomas D. (2002). The Sealing Ability and Retention Characteristics of Mineral Trioxide Aggregate in a Model of Apexification. *Journal of Endodontics*.

[27] Gharib S. R., Tordik P. A., Imamura G. M., Baginski T. A., Goodell G. G. (2007). A Confocal Laser Scanning Microscope Investigation of the Epiphany Obturation System. *Journal of Endodontics*.

[28] Ordinola-Zapata R., Bramante C. M., Bernardineli N. (2009). A Preliminary Study of the Percentage of Sealer Penetration in Roots Obturated With the Thermafil and RealSeal-1 Obturation Techniques in Mesial Root Canals of Mandibular Molars. *Oral Surgery, Oral Medicine, Oral Pathology, Oral Radiology & Endodontics*.

[29] Bani M., Sungurtekin-Ekçi E., Odabaş M. E. (2015). Efficacy of Biodentine as an Apical Plug in Nonvital Permanent Teeth With Open Apices: An In Vitro Study. *BioMed Research International*.

[30] Martin R. L., Monticelli F., Brackett W. W. (2007). Sealing Properties of Mineral Trioxide Aggregate Orthograde Apical Plugs and Root Fillings in an In Vitro Apexification Model. *Journal of Endodontics*.

[31] Valois C. R., Costa E. D. (2004). Influence of the Thickness of Mineral Trioxide Aggregate on Sealing Ability of Root-End Fillings In Vitro. *Oral Surgery, Oral Medicine, Oral Pathology, Oral Radiology & Endodontics*.

[32] Khullar S., Aggarwal A., Chhina H., Kaur T., Sharma M., Bala D. (2021). Sealer Penetration in the Dentinal Tubules: A Confocal Laser Scanning Microscopy Study. *Endodontology*.

[33] Mandava J., Arikatla S., Chalasani U., Yelisela R. K. (2018). Interfacial Adaptation and Penetration Depth of Bioceramic Endodontic Sealers. *Journal of Conservative Dentistry*.

[34] Nanjappa A. S., Ponnappa K. C., Nanjamma K. K., Ponappa M. C., Girish S., Nitin A. (2015). Sealing Ability of Three Root-End Filling Materials Prepared Using an Erbium: Yttrium Aluminium Garnet Laser and Endosonic Tip Evaluated by Confocal Laser Scanning Microscopy. *Journal of Conservative Dentistry*.

[35] Kokate S. R., Pawar A. M. (2012). An In Vitro Comparative Stereomicroscopic Evaluation of Marginal Seal Between MTA, Glass Inomer Cement & Biodentine as Root End Filling Materials Using 1% Methylene Blue as Tracer. *Endodontology*.

[36] Radeva E., Uzunov T., Kosturkov D. (2014). Microleakage Associated With Retrograde Filling After Root End Resection (In Vitro Study). *Journal of IMAB-Annual Proceeding (Scientific Papers)*.

[37] Ravichandra P. V., Vemisetty H., Deepthi K. (2014). Comparative Evaluation of Marginal Adaptation of Biodentine™ and Other Commonly Used Root End Filling Materials-An Invitro Study. *Journal of Clinical and Diagnostic Research: Journal of Clinical and Diagnostic Research*.

[38] Raskin A., Eschrich G., Dejou J., About I. (2012). In Vitro Microleakage of Biodentine as a Dentin Substitute Compared to Fuji II LC in Cervical Lining Restorations. *The Journal of Adhesive Dentistry*.

[39] Caron G., Azérad J., Faure M. O., Machtou P., Boucher Y. (2014). Use of a New Retrograde Filling Material (Biodentine) for Endodontic Surgery: Two Case Reports. *International Journal of Oral Science*.

[40] Bansal R., Bansal M., Matta M. S., Walia S., Kaur B., Sharma N. (2019). Evaluation of Marginal Adaptation of MTA, Biodentine, and MTA Plus as Root-End Filling Materials—An SEM Study. *Dental Journal of Advance Studies*.

[41] Puppala R., Kethineni B., Raghavendra K. J., Abbas A., Birapu U. C., Reddy P. (2020). Efficacy of Mineral Trioxide Aggregate and Biodentine as Apical Barriers in Immature Permanent Teeth: A Microbiological Study. *International Journal of Clinical Pediatric Dentistry*.

[42] Tolibah Y. A., Kouchaji C., Lazkani T., Ahmad I. A., Baghdadi Z. D. (2022). Comparison of MTA Versus Biodentine in Apexification Procedure for Nonvital Immature First Permanent Molars: A Randomized Clinical Trial. *Children*.

[43] Tek G. B., Keskin G. (2021). Use of Mineral Trioxide Aggregate With or Without a Collagen Sponge as an Apical Plug in Teeth With Immature Apices. *Journal of Clinical Pediatric Dentistry*.

[44] Jho W., Park J. W., Kim E. (2016). Comparison of Root Canal Filling Quality by Mineral Trioxide Aggregate and Gutta Percha Cones/AH Plus Sealer. *Dental Materials Journal*.

[45] Butt N., Talwar S., Chaudhry S., Nawal R. R., Yadav S., Bali A. (2014). Comparison of Physical and Mechanical Properties of Mineral Trioxide Aggregate and Biodentine. *Indian Journal of Dental Research*.

[46] Tomer A. K., Miglani A., Nagarjuna P., Chauhan P., Rana S., Vaidya S. (2017). Clinical, Radio Graphical Evaluation of Efficacy of Revascularization by Using Mineral Trioxide Aggregate and Biodentine With Triple Antibiotic Paste in Immature Permanent Incisor Teeth: An In Vivo Study. *International Journal of Applied Decision Sciences*.

[47] Han L., Okiji T. (2011). Uptake of Calcium and Silicon Released From Calcium Silicate–Based Endodontic Materials Into Root Canal Dentine. *International Endodontic Journal*.

[48] Sreedev C. (2017). Comparative Evaluation of Pushout Bond Strength and Apical Sealing Ability of Three Different Root Canal Sealers: An In vitro Study. *Journal of Clinical and Diagnostic Research*.

[49] Singh R., Pushpa S., Arunagiri D., Sawhny A., Misra A., Sujatha R. (2016). The Effect of Irrigating Solutions on the Apical Sealing Ability of MTA Fillapex and Adseal Root Canal Sealers. *Journal of Dental Research, Dental Clinics, Dental Prospects*.

[50] Ballullaya S. V., Vinay V., Thumu J., Devalla S., Bollu I. P., Balla S. (2017). Stereomicroscopic Dye Leakage Measurement of Six Different Root Canal Sealers. *Journal of Clinical and Diagnostic Research: Journal of Clinical and Diagnostic Research*.

[51] Saraswathi D. D., Tejavath S. K., Babu M. R., Swetha B., Gandhi B. (2015). A Comparative Evaluation of Sealing Ability of Three Recent Root-End Filling Materials: An In Vitro Study. *Journal of Advanced Oral Research*.

[52] AlHabdan A. A. (2017). Review of Microleakage Evaluation Tools. *Journal of International Oral Health*.

[53] Raskin A., d’Hoore W., Gonthier S., Degrange M., Dejou J. (2001). Reliability of In Vitro Microleakage Tests: A Literature Review. *The Journal of Adhesive Dentistry*.

[54] Taylor M., Lynch E. (1992). Microleakage. *Journal of Dentistry*.

[55] Raghavendra S. S., Jadhav G. R., Gathani K. M., Kotadia P. (2017). Bioceramics in Endodontics–A Review. *Journal of Istanbul University Faculty of Dentistry*.

[56] Gandolfi M. G., Iezzi G., Piattelli A., Prati C., Scarano A. (2017). Osteoinductive Potential and Bone-Bonding Ability of ProRoot MTA, MTA Plus and Biodentine in Rabbit Intramedullary Model: Microchemical Characterization and Histological Analysis. *Dental Materials*.

[57] Sultana N., Singh M., Nawal R. R. (2018). Evaluation of Biocompatibility and Osteogenic Potential of Tricalcium Silicate–Based Cements Using Human Bone Marrow–Derived Mesenchymal Stem Cells. *Journal of Endodontics*.

