# Supplementary material for: Evaluation of Microleakage of Orthograde Root-Filling Materials in Immature Permanent Teeth: An In Vitro Study
Source: Int J Biomater. 2024 Oct 29;2024:8867854. doi: 10.1155/2024/8867854 (PMC11537741; doi:10.1155/2024/8867854)
Supplement: Supporting Information 1 — Supporting document 1: Figure 1: Sample collection and group division, Figure 2: Creation of divergent apex and root canal preparation, and Figure 3: Radiographic image of sample showing divergent apex. [file 8867854.f1.docx]

**
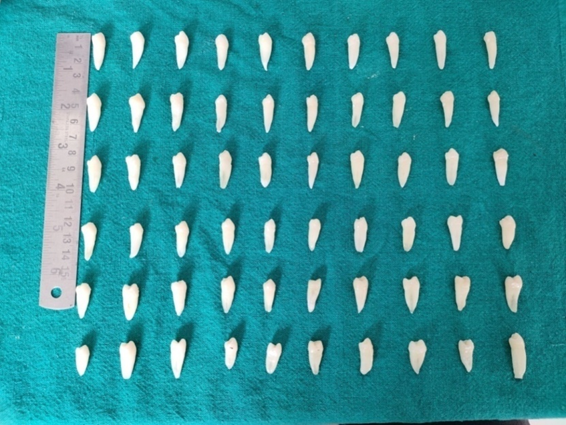
 Supplementary document 1**


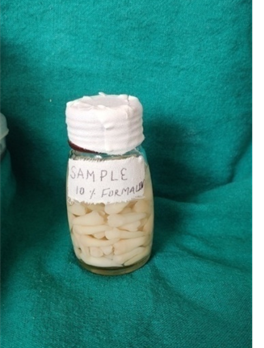


Figure 1: Sample collection and group division


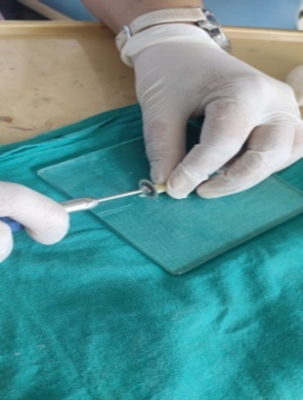


Figure 2: Creation of divergent apex and root canal preparation


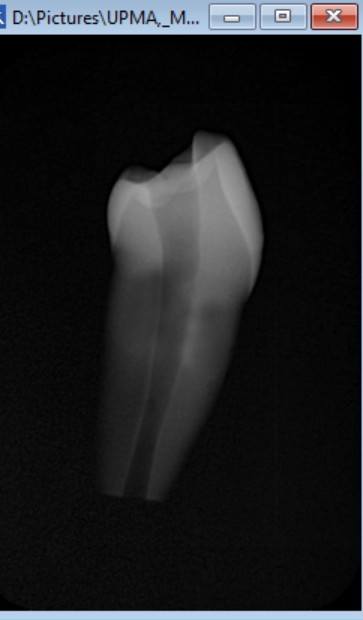


Figure 3: Radiographic image of sample showing divergent apex
