# Supplementary material for: Evaluation of Microleakage of Orthograde Root-Filling Materials in Immature Permanent Teeth: An In Vitro Study
Source: Int J Biomater. 2024 Oct 29;2024:8867854. doi: 10.1155/2024/8867854 (PMC11537741; doi:10.1155/2024/8867854)
Supplement: Supporting Information 2 — Supporting document 2: Figure 1: Samples with MTA plug, Figure 2: Samples with Biodentine plug, Figure 3: Samples with MTA plus sealer with GP, Figure 4: Samples with Adseal sealer with GP (Group IV), Figure 5: Negative control group, and Figure 6: Positive control group. [file 8867854.f2.docx]

**Supplementary document 2**


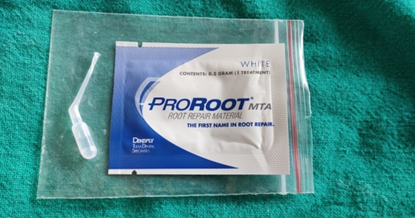

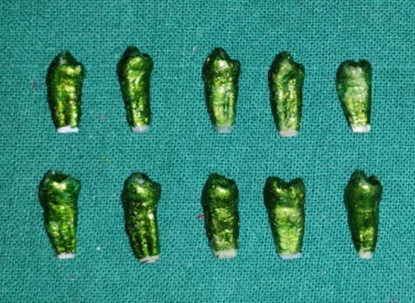


Figure 1: Samples with MTA plug


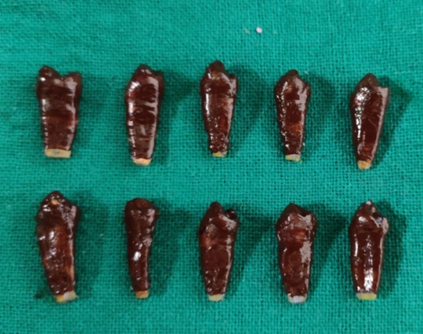


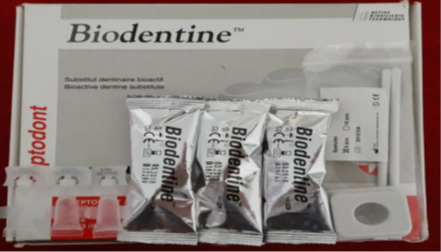


Figure 2: Samples with Biodentine plug


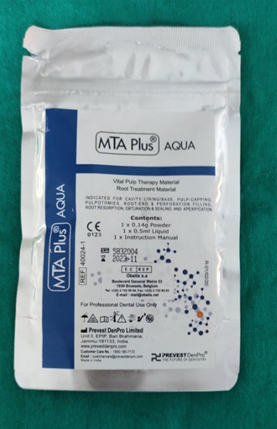

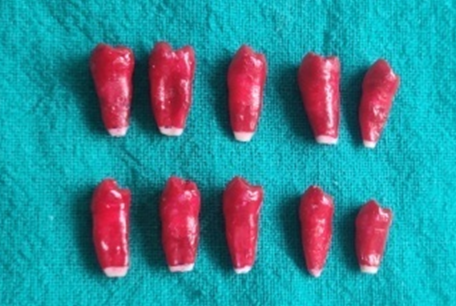


Figure 3: Samples with MTA plus sealer with GP


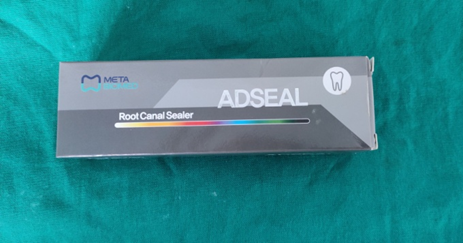

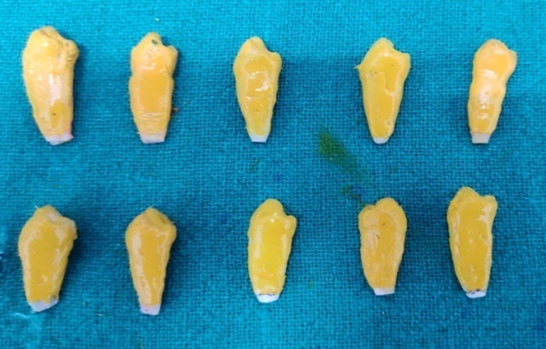


Figure 4: Samples with Adseal sealer with GP (Group IV)


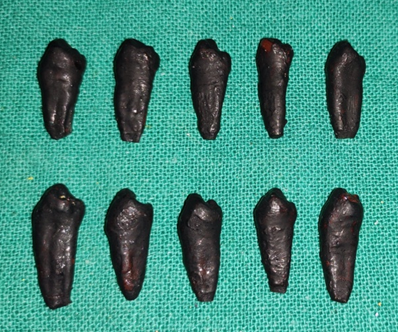

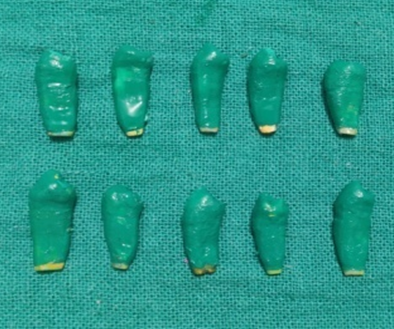


Figure 5: Negative control group Figure 6: Positive control group
