# Supplementary material for: Evaluation of Microleakage of Orthograde Root-Filling Materials in Immature Permanent Teeth: An In Vitro Study
Source: Int J Biomater. 2024 Oct 29;2024:8867854. doi: 10.1155/2024/8867854 (PMC11537741; doi:10.1155/2024/8867854)
Supplement: Supporting Information 3 — Supporting document 3: Figure 1: Immersion of samples in 2% Rhodamine dye and Figure 2: Fluorescence microscope used for examination of sample cross sections. [file 8867854.f3.docx]

**Supplementary document 3**


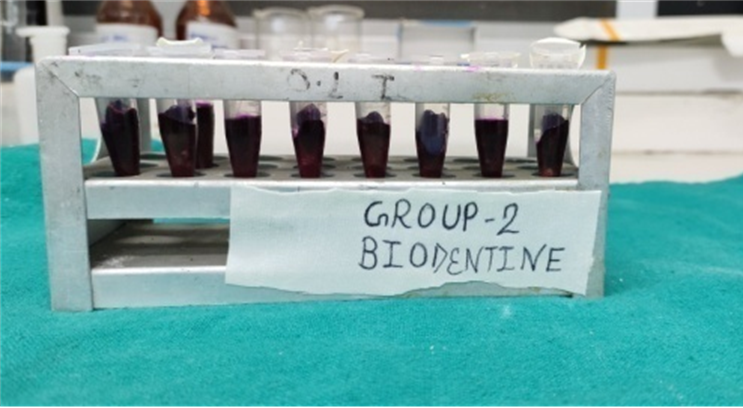


Figure 9: Immersion of samples in 2% Rhodamine dye


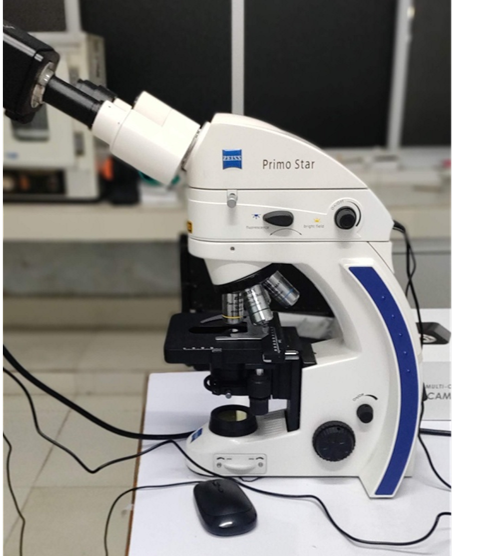


Figure 10: Fluorescence microscope used for examination of sample cross-sections
