# Supplementary material for: Evaluation of Microleakage of Orthograde Root-Filling Materials in Immature Permanent Teeth: An In Vitro Study
Source: Int J Biomater. 2024 Oct 29;2024:8867854. doi: 10.1155/2024/8867854 (PMC11537741; doi:10.1155/2024/8867854)
Supplement: Supporting Information 4 — Supporting document 4: Materials. [file 8867854.f4.docx]

| MATERIALS | (LOT NUMBERS) | MANIPULATION TECHNIQUE |
| --- | --- | --- |
| 1.Biodentine | B16765, B16765 | Biodentine™ was prepared following the specified mixing instructions outlined in the Instructions for Use (IFU). The process involved taking the Biodentine™ ampoule and positioning it in the manufacturer-provided stand. Subsequently, the ampoule lid was opened, and five drops of liquid were introduced. After closing the Biodentine™ lid, the ampoule underwent mixing in the machine for a duration of 30 seconds. |
| 2. Pro Root MTA | Not available | To initiate the preparation process, begin by opening a pouch of ProRoot® MTA root repair material and empty the powder onto a mixing pad. Following this, remove the end of a ProRoot® liquid micro-dose ampoule and release its contents onto the mixing pad adjacent to the root repair material. Gradually incorporate the liquid into the cement using the ProRoot® MTA mixing stick. Ensure a thorough blend by mixing the material with the liquid for about one minute, allowing complete hydration of all powder particles as per manufacturer instructions. |
| 3.MTA PLUS (PERVEST) | 5832004 | As per manufracturers instructions, start by dispensing one scoop of powder onto a non-absorbent pad or glass slab. Place a small drop of gel or MTAPlus™ Liquid from one ampoule next to the powder. Gradually mix the liquid or gel into the powder until achieving the desired putty-like consistency, or for some procedures, a thinner syrupy, stringy consistency. Ensure thorough mixing to hydrate the powder. If not using the material immediately, cover it with a moist gauze sponge or a clean cover to prevent evaporation. Extra gel or MTA Plus™ liquid can be applied to rehydrate the powder before it sets. |
| 4.ADseal sealer (META) | Not available | As per manufacturer instructions, combine base and catalyst in a 2:1 weight ratio on a mixing pad using a spatula. Mix thoroughly for 15-20 seconds until a creamy and homogeneous consistency is achieved. This ensures an effective blend for optimal performance in subsequent applications. |
